# Mapping antimicrobial resistance awareness in Canada: what are our knowledge gaps?

**DOI:** 10.1093/jacamr/dlag162

**Published:** 2026-08-26

**Authors:** Helena Ferreira Leal, Fazia Tadount, Jie He, Élise Fortin, Françoise Bichai, Caroline Quach, Emilie Bédard

**Affiliations:** Department of Microbiology, Infectious Diseases, and Immunology, Faculty of Medicine, Université de Montréal (UdeM), Montréal, Québec, Canada; Department of Microbiology, Infectious Diseases, and Immunology, Faculty of Medicine, Université de Montréal (UdeM), Montréal, Québec, Canada; Centre Hospitalier Universitaire Sainte-Justine (CHU Sainte-Justine), Montréal, Québec, Canada; Department of Economy, Université de Sherbrooke (USherbrooke), Sherbrooke, Québec, Canada; Department of Microbiology, Infectious Diseases, and Immunology, Faculty of Medicine, Université de Montréal (UdeM), Montréal, Québec, Canada; Institut National de Santé Publique du Québec (INSPQ), Québec, Québec, Canada; Department of Civil, Geological and Mining Engineering, Polytechnique Montréal, Montréal, Québec, Canada; Department of Microbiology, Infectious Diseases, and Immunology, Faculty of Medicine, Université de Montréal (UdeM), Montréal, Québec, Canada; Centre Hospitalier Universitaire Sainte-Justine (CHU Sainte-Justine), Montréal, Québec, Canada; Department of Civil, Geological and Mining Engineering, Polytechnique Montréal, Montréal, Québec, Canada

## Abstract

**Background:**

Often referred to as a silent pandemic, antimicrobial resistance (AMR) is gradually expanding worldwide. Addressing this escalating threat requires collaborative efforts from governments, stakeholders and community members; thus, fostering widespread public knowledge is crucial. We aimed to assess the knowledge, attitudes and practices (KAPs) related to AMR in a diverse cohort from the province of Quebec, Canada, to identify critical gaps in targeted educational interventions.

**Methods:**

An observational, cross-sectional, descriptive study was conducted from April 2022 to August 2023. Participants included individuals 18 years or older from urban areas in Quebec, focusing on the cities of Montreal and Laval. The data were collected using a questionnaire adapted from the World Health Organization’s ‘Antibiotic Resistance: Multi-country Public Awareness Survey’.

**Results:**

The study engaged 1774 participants and revealed a fair understanding of AMR, with misconception rates varying from 9.9% to 58.3%. However, significant knowledge gaps were identified. When asked about following the recommended length of treatment and daily dosage of antibiotics, 13.8% affirmed not fully adhering. When asked if medication was stopped once symptoms started to disappear, 7.3% responded ‘always’ and 20.4% ‘sometimes’. Additionally, 12.5% reported taking oral antibiotics without a prescription. Notably, men, younger audiences (18–34) and individuals with lower formal education were more prone to risky AMR-related behaviours.

**Conclusions:**

This study reveals significant gaps in public understanding and practices related to AMR in Quebec. Enhanced public education campaigns, stricter antimicrobial use regulations and tailored educational programmes targeting high-risk groups are recommended. An improved understanding of AMR can significantly contribute to global efforts to combat this pressing health issue.

## Introduction

Antimicrobial resistance (AMR) refers to the ability of microorganisms to withstand the effects of antimicrobial agents, leading to treatment failures and increased morbidity and mortality.^1,2^ Historically, the high levels of resistance encountered in clinical and community settings were primarily attributed to the overuse and misuse of antimicrobial agents in human medicine.^3,4^ However, AMR is now recognized as a multifaceted phenomenon arising from complex interactions between humans, animals and the environment.^5,6^ Consequently, tackling this threat demands multisectoral interventions and a joint effort involving governments, stakeholders and community members.^7,8^

In the community, the spread of AMR is facilitated through various channels, including person-to-person contact, interactions with animals (from pets to occupational exposure) and dissemination via environmental matrices, particularly water.^9^ Humans and animals shed resistant bacteria, AMR genes and antimicrobial residues in water through their bodily fluids and excrement.^10–12^

An informed community can significantly contribute to mitigating the spread of AMR by understanding the impact of their actions on environmental health.^13^ This awareness ultimately leads individuals to adopt responsible antimicrobial usage practices, such as using antibiotics only when prescribed by a healthcare professional, properly disposing of unused medication, avoiding the misuse of antimicrobials in pets and limiting transmission of infectious diseases through measures such as vaccination.^14^ Also, compliance with water, sanitation and hygiene (WASH) measures is critical to combating AMR.^9^ Moreover, empowered communities can actively demand and support government initiatives to combat AMR.

The World Health Organization (WHO)^15^ and the Public Health Agency of Canada^8^ have identified public awareness as a cornerstone of AMR mitigation and a key objective of the Global Action Plan.^7^ In 2015, the ‘Antibiotic Resistance: Multi-country Public Awareness Survey’ conducted by the WHO revealed critical gaps in public understanding and practices related to antibiotic use, underscoring the need for targeted educational and awareness-raising interventions.^16^ Despite ongoing efforts to address AMR in Canada, there is still a significant gap in comprehensively understanding how the general population perceives and responds to this threat, with few studies on this specific topic.^17–19^

Understanding public perceptions of AMR is particularly important given the growing burden of AMR in Canada. National data indicate that approximately 26% of documented bacterial infections were resistant to first-line antimicrobials in 2018, a proportion projected to reach 40% by 2050.^20^ According to current projections, AMR could result in up to 13 700 deaths annually and a cumulative economic burden of $388 billion dollars.^20^

In 2022, we conducted a study focused on understanding the knowledge, attitudes, and practices (KAPs) of the student population in Montreal regarding AMR.^21^ More than half of participants were unaware of the actual causes of resistance, mistakenly believing that AMR occurs when their body becomes resistant to antimicrobials rather than bacteria. Our current research broadened our focus to the general population, targeting residents of two major Canadian cities in the province of Quebec, Canada. Our aim is to identify specific knowledge and practice gaps, allowing policymakers and public health officials to develop targeted interventions to combat AMR in the Canadian context, such as educational initiatives, regulatory reforms and community engagement strategies.

## Methodology

### Study design and population

We conducted an observational, cross-sectional, descriptive study, using the Simple Survey platform, which was active from 21 April 21 2022, to 8 August 2023. Our target population consisted of individuals 18 years or older from Quebec, mainly focusing on the urban areas of Montreal and Laval. The exclusion criteria included prior survey participation, household member redundancy, and the systematic provision of incongruent or logically inconsistent responses.

### Data collection

We designed a questionnaire adapted from the WHO’s ‘Antibiotic Resistance: Multi-country Public Awareness Survey’.^16^ Using a non-probabilistic convenient sampling strategy, we disseminated the survey via Meta's platforms, given its extensive reach and prior to any restrictions imposed by governmental bodies. Additionally, we used electronic mailing lists of student associations, university departments and research centres within the investigators’ professional networks. The survey was available in French and English. Participation was anonymous and voluntary.

The survey comprised 40 questions to evaluate the respondents’ knowledge of AMR, its theoretical basis, and their perceptions of appropriate measures to address this health issue. We also explored lifestyle behaviours potentially contributing to AMR, such as cooking and hygiene habits, contact with animals, and antibiotic usage practices. The complete questionnaire is available in the Table S1 (available as Supplementary data at *JAC-AMR* Online).

### Data management and analysis

Data management and analysis were performed using RStudio version 4.1.2, and charts were created using Microsoft Excel 2010. We conducted univariate analyses to identify groups with a greater likelihood of incorrect KAPs related to AMR, focusing on statements with misconception rates above 5%. We assessed statistical significance using chi-square or Fisher’s exact tests, as appropriate.

We conducted logistic regression to further examine factors associated with misconceptions. We included variables that showed significant associations in univariate analyses, as well as *a priori* confounders. Text S2 provides additional details regarding statistical analyses and model selection. The detailed R script, dataset used for this analysis, and a data dictionary are also available in the Supplementary material.

## Results

We disseminated our survey through mailing lists and various social media platforms (Text S1). Out of 2081 individuals who participated in the survey, 1821 provided complete and valid answers (87.5% completion rate), resulting in 1774 remaining participants after applying exclusion criteria. Detailed participant selection and exclusion criteria can be found in Figure S1.

### Demographic profile of the study sample

The study population was primarily women and Canadian citizens (Table 1). Participation was distributed across the different age groups. Participants had a broad spectrum of occupational and educational backgrounds, with health-related fields and areas such as commerce, administration and economics being the most prevalent, with a significant percentage having completed undergraduate or graduate studies. Most participants were residents of urban areas or suburbs, with a significant concentration in Montreal and Laval (Table S1).

**Table 1.** Sociodemographic characteristics

Demographics	Our sample		Quebec’s population
	*n* = 1774	%	%
Gender^a^			
Women	1227	69.2	50.6
Men	490	27.6	49.4
Other	29	1.6	—
Age^b^			
18–24	143	8.1	8.2
25–34	380	21.4	14.6
35–44	372	21.0	13.5
45–54	211	11.9	12.9
55–64	304	17.1	15.0
65+	343	19.3	20.4
Nationality^c^			
Canadian	1469	82.8	91.4
Other	74	4.2	8.6
Formal education level^d^			
No formal education	20	1.1	5.6
Primary school	69	3.9	6.7
General secondary (DES)	171	9.6	15.0
Secondary technical school (DEP)	166	9.4	12.1
General college (CEGEP)	165	9.3	10.2
Technical college (CEGEP)	310	17.5	17.6
Bachelor's degree	455	25.6	24.6
Graduate studies	378	21.3	8.2
Current employment status^e^			
Employee	633	35.7	49.5
Retired	501	28.2	20.3
Student	254	14.3	10.1
Independent worker/self-employed	136	7.7	10.7
Unemployed	94	5.3	7.4
Unable to work due to medical reasons	92	5.2	2.8
Other	27	1.5	0.6
Field of work or study^f^			
Agriculture, agro-food, environment	99	5.6	2.2
Arts, culture	145	8.2	3.9
Construction	157	8.9	6.2
Commerce, management, economy, administration	255	14.4	18.0
Law, political science, security	127	7.2	3.5
Education, teaching	253	14.3	7.7
Hotels, restaurants, tourism	109	6.1	6.8
Industry	92	5.2	7.8
Information, communication	138	7.8	7.9
Literature, languages, humanities	107	6.0	2.5
Health	438	24.7	11.6
Science	226	12.7	3.6
Social	116	6.5	4.8
Sport, entertainment	40	2.3	1.3
Transport, logistics	42	2.4	3.5
Other	128	7.2	1.9
Location^g^			
Urban—in a highly populated city (opposite to rural)	914	51.5	51.3
Suburbs—on the outskirts of a city	724	40.8	37.5
Rural—outside a city, such as a village, countryside, or agricultural area	218	12.3	11.2
City^h^			
Montreal and other cities within Montreal Island	666	37.5	42.7
Laval	192	10.8	5.1
Longueuil	52	2.9	2.8
Repentigny	26	1.5	1.0
Saint Jerome	22	1.2	0.9
Blainville	22	1.2	0.7
Terrebonne	21	1.2	1.4
Other cities in Québec	378	21.3	45.4
Type of residence^j^			
Apartment	831	46.8	45.1
House (individual)	558	31.5	44.3
Joint tenancy	288	16.2	7.2
Other	36	2.0	3.4
Number of bedrooms in the residence^i^			
1–2 bedrooms	738	41.6	39.4
3–4 bedrooms	860	48.5	47.6
≥5 bedrooms	125	7.0	6.0
Household composition (non-mutually exclusive options)			
Babies (aged ≥0 to ≤ 1)	222	12.5	10.2
Toddlers (aged ≥1 to ≤ 3)	267	15.1	14.1
Children (aged ≥4 to ≤ 10)	374	21.1	19.4
Preteens (aged ≥11 to ≤ 14)	327	18.4	17.0
Teenagers (aged ≥15 to ≤ 19)	368	20.7	18.3
Adults (aged ≥20 to ≤ 65)	1380	77.8	76.5

The sociodemographic characteristics of the 1774 participants included in the study are shown in the table. The data regarding the population of Quebec were obtained from the 2021 Census,^22^ available on the Statistics Canada website. Categories were grouped to facilitate comparison between the study data and the general population of Quebec. To evaluate whether differences in KAPs existed among the various groups, respondents were segmented by gender, age, professional/academic field, nationality and education level (Tables 2–5). Perceptions with a misconception frequency greater than 5% were included in this univariate analysis. Abbreviations: CEGEP, collège d'enseignement général et professionnel; DEP, diplôme d'études professionnelles; DES, diplôme d'études secondaires.

^a^Twenty-eight (1.57%) participants did not wish to specify their gender.

^b^Twenty-one (1.18%) participants did not wish to specify their age.

^c^Two hundred thirty-one231 (13.02%) participants did not wish to specify their citizenship.

^d^Forty (2.25%) participants did not wish to specify their level of education.

^e^Thirty-seven (2.08%) participants did not wish to specify their professional status.

^f^Seventy-four (4.17%) participants did not wish to specify their type of professional activity.

^g^Thirty-eight (2.14%) participants did not wish to specify their location.

^h^Three hundred ninety-five participants did not wish to specify in which city they lived.

^i^Fifty-one (2.87%) participants did not wish to specify the number of bedrooms in their residences.

^j^Sixty-one (3.43%) participants did not wish to specify their type of residence.

### Knowledge, attitudes, and practices

#### Antimicrobial use

**Figure 1.**
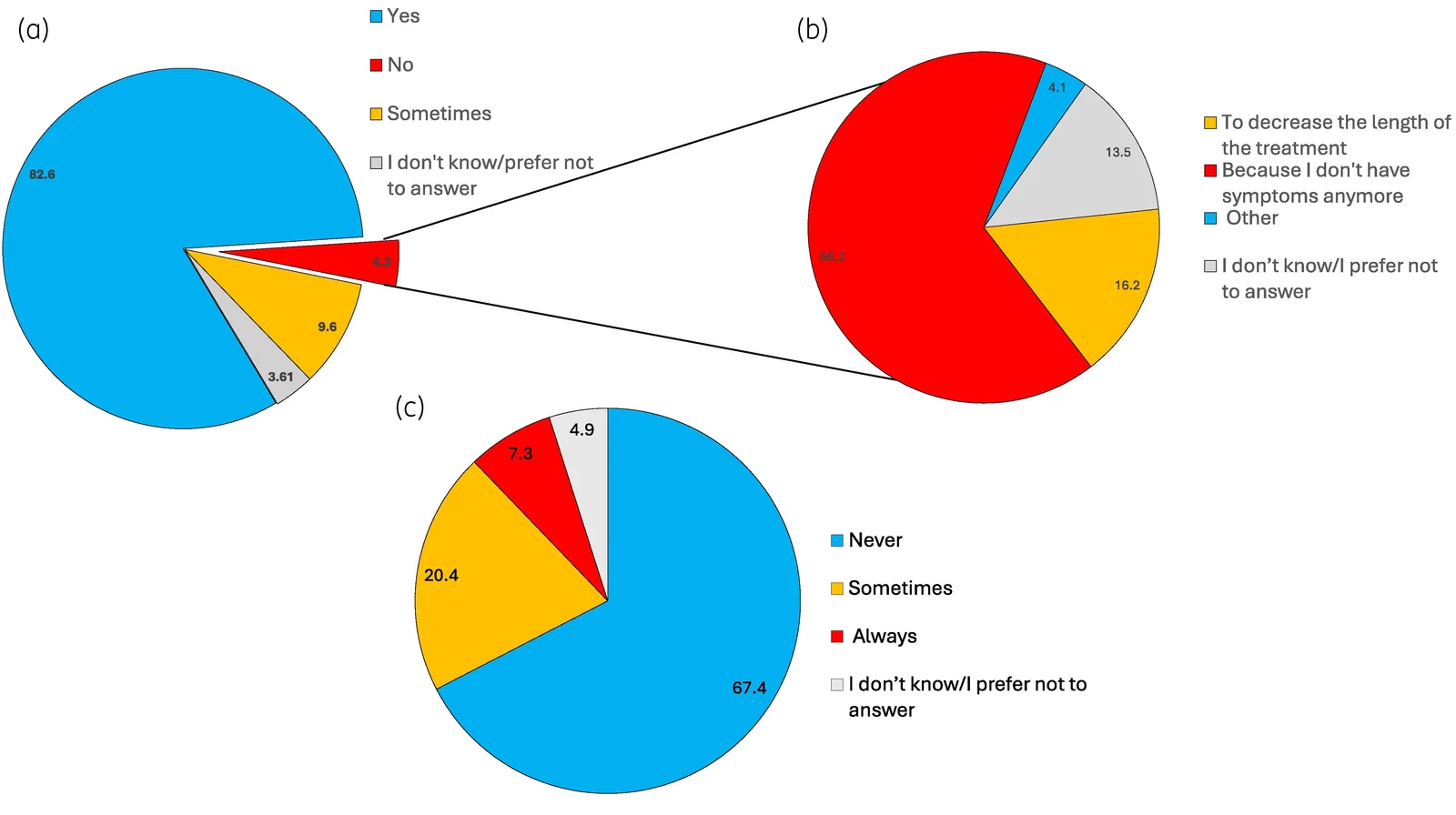
Self-reported adherence to antimicrobial treatment regimens (*n* = 1774). (a) Visualizes participant adherence to prescribed treatment duration and dosage, with "Yes" indicating compliance, "Sometimes" for occasional non-compliance, "No" for regular non-compliance and grey for those uncertain or choosing not to answer. In (b), reasons for non-adherence are shown, with "Because I don′t have symptoms anymore" highlighting cessation of antibiotics once symptoms disappear, "To decrease the length of the treatment" for shortening the treatment duration and blue for other reasons. "I don′t know/I prefer no to answer" captures the respondents who were uncertain or did not answer. (c) Depicts the frequency of stopping medication with symptom resolution. "Never"signifies those who do not stop taking medication prematurely, "Sometimes" for those who sometimes do and "Always" for those who always stop once feeling better. "I don′t know/ I prefer not to answer" includes uncertain respondents or those who preferred not to disclose their behaviour.

Our findings indicated a high level of adherence to antimicrobial treatment regimens, with most participants following the prescribed length of treatment and daily dosage (Figure 1a). However, deviations from recommended protocols occurred: 4.2% did not consistently adhere, and 9.6% sometimes failed to follow medical advice.

Among non-adherent participants, the cessation of symptoms was the predominant reason for incomplete treatment (Figure 1b). Additionally, a significant portion of respondents sometimes discontinued medication once symptoms improved (Figure 1c), and 7.3% always stopped their medication prematurely.

**Figure 2.**
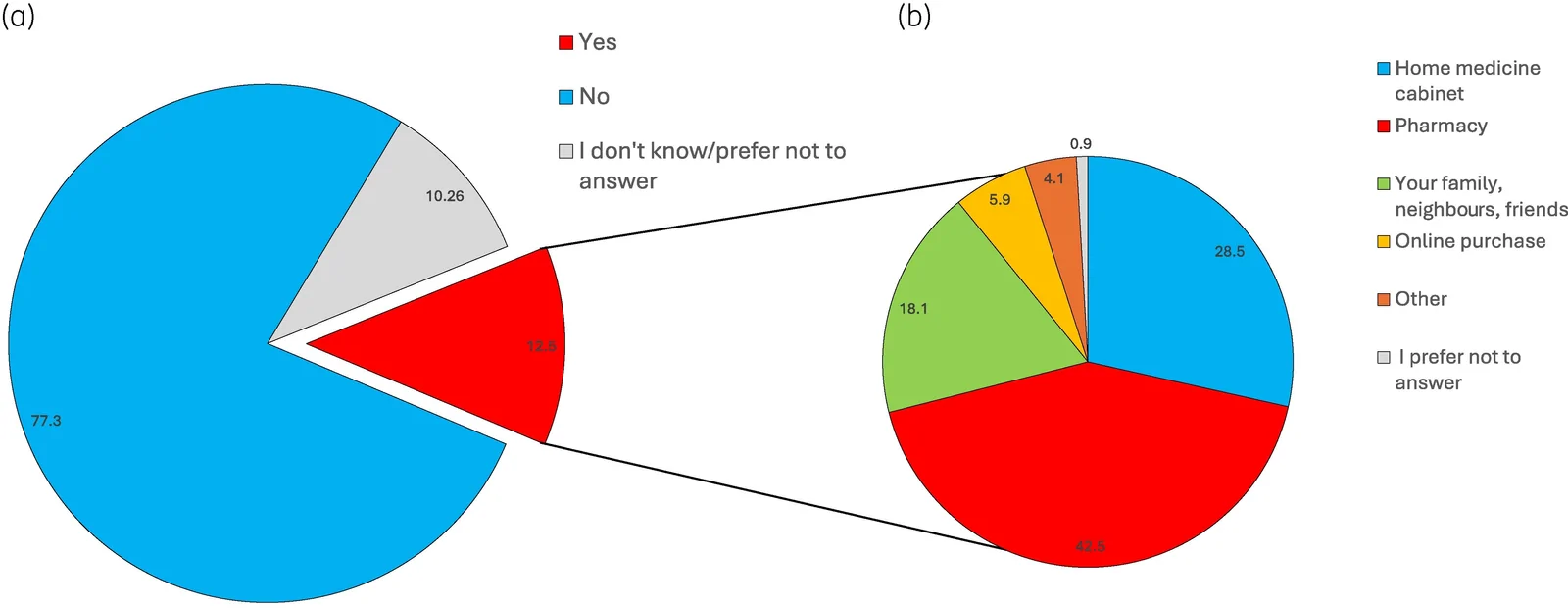
Unprescribed antibiotic use and sources (*n* = 1774). (a) Depicts participant responses to whether they have ever taken oral antibiotics without a prescription. The "No" section represents those who have not, the "Yes" portion indicates those who have and the "I don′t know/prefer not to answer" slice covers those who are uncertain or prefer not to answer. In (b), the slices categorize the various sources from which participants obtained antibiotics without a prescription. The "Pharmacy" section is the largest, showing that most obtained antibiotics from these establishments. Antibiotics from family, neighbours, or friends represent 18.1%; those from the home medicine cabinet 28.5%; online purchases 4.1%; and other unspecified sources 5.9%. The smallest section accounts for respondents who did not answer or were unsure (0.9%).

Our data suggest that non-prescribed antimicrobial use occurred in a segment of the population (Figure 2a), raising concerns about informal access to these drugs. Specifically, 12.5% of respondents said that they took antibiotics without a prescription.

When asked about the sources of non-prescribed antimicrobials (Figure 2b), participants identified pharmacies as the primary outlet (42.5%), followed by their home medicine cabinets (28.5%). Antimicrobials were also obtained through family, neighbours or friends (18.1%) and online purchases (5.9%). Less frequent sources included self-prescription, purchases made abroad and veterinary prescriptions.

#### AMR awareness

Participants demonstrated varying levels of awareness of key terms associated with AMR. The recognition of these AMR-related terms was substantial, with familiarity proportions ranging from 51.5% to 84.4% (Figure S2). However, ‘antimicrobial resistance’ was known by only 41% of participants.

Most participants in our study learned about these terms from traditional media, followed by the internet, including social media (Figure S3). Nearly half of participants were informed by healthcare professionals. However, specific campaigns appeared to have a more limited impact.

**Figure 3.**
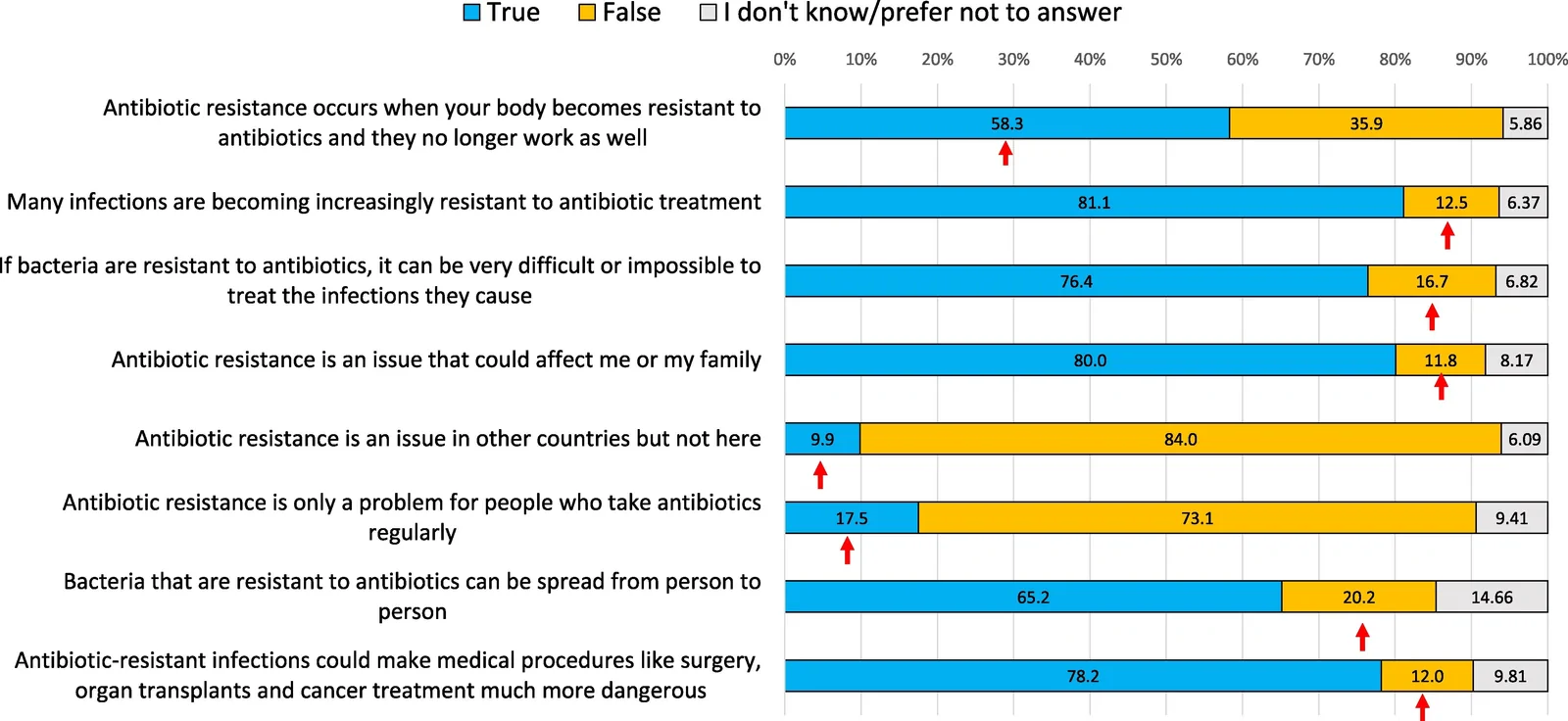
General knowledge on AMR and its impact (*n* = 1774). The chart delineates respondents’ understanding of statements about AMR’s theoretical basis and impact. Statements are listed along the vertical axis, with corresponding response percentages along the horizontal axis. For each statement, the left, middle, and right segments of the horizontal bar represent “True,” “False,” and “I don’t know/prefer not to answer” responses, respectively. Arrows underscore the percentage of incorrect beliefs.

The analysis of participants’ general knowledge of AMR revealed gaps in their understanding of its fundamental principles (Figure 3). Misconception proportions varied from 9.9% to 58.3%, depending on the topic. A significant proportion of participants (58.3%) mistakenly believed that antibiotic resistance occurred when their bodies became resistant to the drugs.

Furthermore, some respondents erroneously considered antibiotic resistance to be an issue solely affecting those who take antibiotics frequently, while 20.2% did not acknowledge that resistant bacteria can spread from person to person. Moreover, 9.9% of the respondents erroneously perceived AMR as a problem restricted to countries outside of Canada.

#### AMR across the One Health spectrum

Participants exhibited diverse levels of understanding regarding the implications of AMR for animal and environmental health, with proportions of misconceptions ranging from 10.8% to 26.3% (Figure S4). Nearly half of our participants had pets. While most pet owners were cautious and limited antibiotic use to the treatment of infections, some administered antibiotics to their animals frequently (Figure S5).

Additionally, some participants were unaware that the use of antibiotics in livestock and crops could contribute to the rise of AMR in the environment, and 26.3% did not recognize the risk of acquiring resistant bacteria themselves, suggesting a disconnect in understanding the full transmission cycle of AMR. Furthermore, 10.8% believed that AMR was confined to hospital settings.

#### Cooking and eating hygiene practices

Most participants adhered to recommended cooking and eating hygiene practices (Figure S6). Although most participants washed their hands before eating, a substantial portion did not. This was also observed for handwashing before cooking. A significant proportion of participants reported not washing fruits and vegetables before consumption (Figure S6).

#### Mitigating AMR

**Figure 4.**
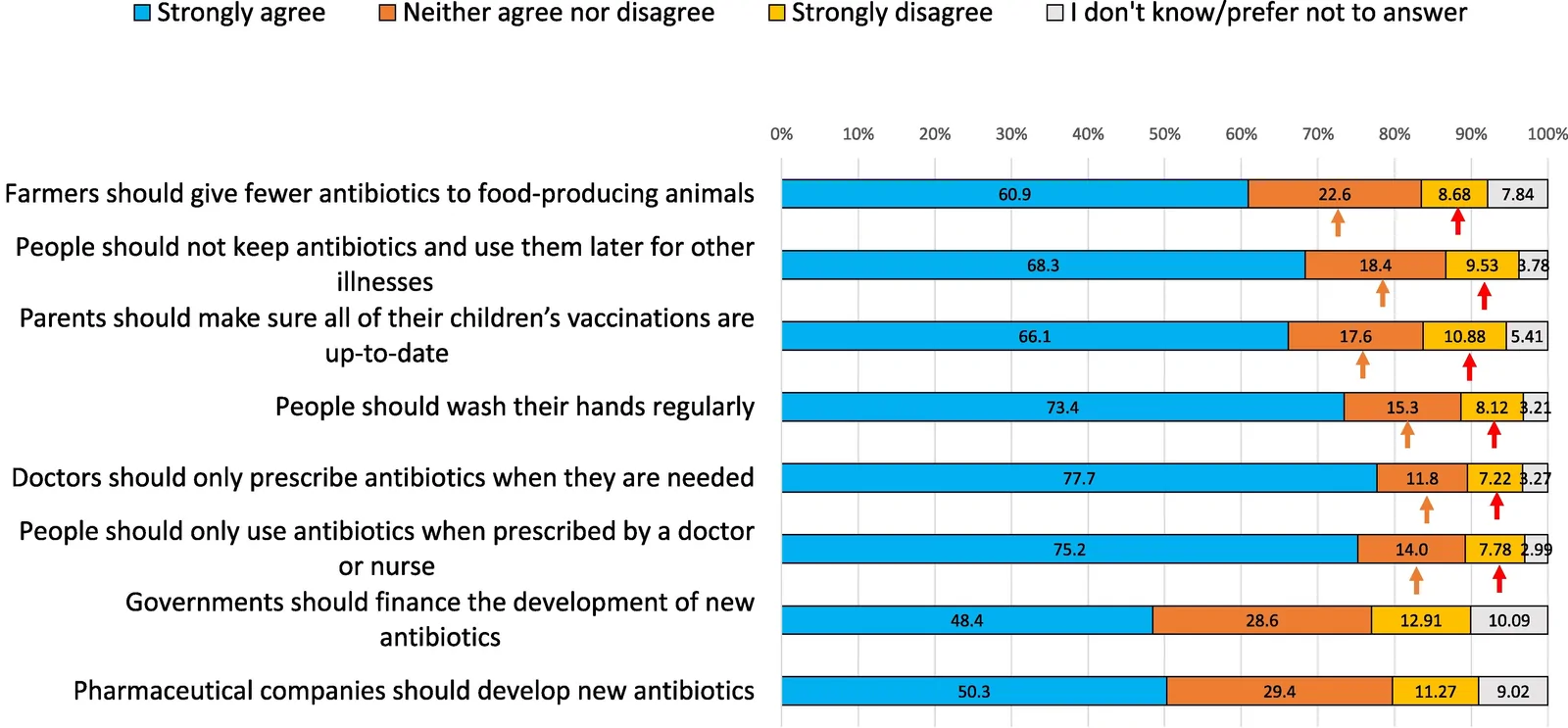
Strategies and policies for combating AMR (*n* = 1774). The bar chart illustrates survey participants’ levels of awareness and understanding of actions taken to combat AMR. Statements are listed along the vertical axis, with corresponding response percentages along the horizontal axis. For each statement, the segments of the horizontal bar, from left to right, represent “Strongly agree,” “Neither agree nor disagree,” “Strongly disagree,” and “I do not know/prefer not to answer,” respectively. Arrows point to significant instances of incorrect beliefs or neutrality.

According to our analysis (Figure 4), 8.7% of respondents disagreed, and 22.6% were undecided about whether farmers should use fewer antibiotics for animals. Regarding the retention and reuse of antibiotics, 9.5% mistakenly believed it was safe, and 18.4% neither agreed nor disagreed with this practice. Uncertainty about vaccinations was also notable: 17.6% of participants were ambivalent, and 10.9% were strongly against the importance of vaccinating children to prevent infections with resistant organisms. Only 73.4% recognized the importance of hand hygiene to mitigate AMR.

Furthermore, opinions were split regarding funding for new antibiotics, with 28.6% being neutral about whether governments should invest in their development and 29.4% were undecided as to the role of pharmaceutical industries.

The analysis (Figure S7) showed that the statement ‘Everyone needs to use antibiotics responsibly’ garnered the highest agreement rate (79%). Most participants disagreed with the notion that they were safeguarded against AMR by using antimicrobials correctly, indicating an understanding that AMR is a complex issue.

However, there was a sense of resignation among some participants about their ability to affect AMR outcomes. As many as 15.3% strongly agreed with the statement ‘People like me cannot do much to stop antibiotic resistance’, while 34.1% of participants remained ambivalent, signalling a potential gap in public empowerment and engagement strategies (Figure S7).

Most participants did not believe that AMR is one of the greatest problems the world currently faces, with 59.9% expressing this sentiment either by disagreement or ambivalence. At the same time, there was widespread scepticism about medical experts’ ability to pre-emptively solve the AMR problem before it becomes too serious.

#### Comparison between groups

We focused our analysis on propositions with a misconception rate exceeding 5%. The results of this exhaustive analysis are detailed in Table 6. Statistically significant associations for misconceptions for selected demographic factors are presented in Tables 2–5.

**Table 2.** Incorrect KAPs related to AMR among different groups: gender

KAPs	Gender		
	Women *n* (%)	Men *n* (%)	OR (95% CI)
Cooking habits			
After handling raw food (meat and vegetables), I wash my hands with soap (No)	148 (12.1)	**100** (**20.6)**	1.88 (1.42–2.48)
I use the same kitchen utensils for handling raw and ready-to-eat foods (Yes)	244 (20.4)	**194** (**40.8)**	2.70 (2.13–3.33)
I wash kitchen utensils that have been used for raw food before using them to prepare other foods (No)	182 (15.2)	**98** (**21.0)**	1.48 (1.13–1.94)
I wash fruits and vegetables before eating them (No)	229 (19.1)	**119** (**25.0)**	1.42 (1.10–1.82)
Antimicrobial use			
When you get a prescription for antibiotics, do you follow the recommended length of treatment and daily dosage? (No or sometimes)	129 (11.6)	**108** (**27.6)**	2.49 (1.88–3.30)
Do you stop using the medication once symptoms start to disappear? (Sometimes or always)	236 (19.8)	**237** (**51.6)**	4.31 (3.42–5.44)
Have you ever taken an oral antibiotic treatment (by mouth) without a medical prescription? (Yes)	125 (11.0)	**87** (**20.7)**	2.13 (1.56 -2.86)
Contact with animals			
I can exchange resistant bacteria with my pet (False)	243 (23.9)	**160** (**37.6)**	1.91 (1.49–2.43)
The use of antibiotics in livestock and crops can increase the presence of resistant bacteria in the environment (False)	129 (11.9)	**113** (**27.2)**	2.77 (2.08–3.67)
The use of antibiotics in livestock and crops can affect me directly (I can get resistant bacteria in my body) (False)	─	─	─
Resistant bacteria are only found in hospitals (True)	87 (7.5)	**96** (**21.9)**	3.45 (2.50 -4.76)
General knowledge about AMR			
Antibiotic resistance occurs when your body becomes resistant to antibiotics and they no longer work as well (True)	**750** (**63.9)**	253 (57.0)	1.34 (1.07–1.67)
Many infections are becoming increasingly resistant to antibiotic treatment (False)	106 (9.1)	**94** (**21.2)**	3.72 (2.68–5.17)
If bacteria are resistant to antibiotics, it can be very difficult or impossible to treat the infections they cause (False)	144 (12.5)	**130** (**29.1)**	3.48 (2.60–4.65)
Antibiotic resistance is an issue that could affect me or my family (False)	91 (7.9)	**97** (**22.6)**	2.94 (2.28–3.78)
Antibiotic resistance is an issue in other countries but not here (True)	76 (6.5)	**86** (**19.7)**	2.84 (2.20–3.66)
Antibiotic resistance is only a problem for people who take antibiotics regularly (True)	166 (14.7)	**132** (**31.0)**	3.84 (2.98–4.96)
Bacteria that are resistant to antibiotics can be spread from person to person (False)	191 (18.4)	**144** (**33.7)**	2.65 (2.09–3.36)
Antibiotic-resistant infections could make medical procedures like surgery, organ transplants and cancer treatment much more dangerous (False)	96 (8.5)	**92** (**21.7)**	3.35 (2.60–4.45)
Levels of awareness and understanding of how to combat AMR (strongly disagree or neither agree nor disagree)			
Farmers should give fewer antibiotics to food-producing animals	343 (30.2)	**197** (**43.9)**	1.81 (1.44–2.27)
People should not keep antibiotics and use them later for other illnesses	246 (20.6)	**220** (**48.1)**	3.58 (2.85–4.52)
Parents should make sure all their children’s vaccinations are up-to-date	295 (30.0)	**182** (**51.6)**	2.01 (1.60–2.53)
People should wash their hands regularly	233 (19.3)	**156** (**34.2)**	2.17 (1.72–2.78)
Doctors should only prescribe antibiotics when they are needed	150 (12.5)	**162** (**35.2)**	3.81 (2.95–4.93)
People should only use antibiotics when prescribed by a doctor or nurse	191 (15.9)	**174** (**37.6)**	3.23 (2.56–4.17)
Everyone needs to use antibiotics responsibly	153 (12.7)	**148** (**32.1)**	3.22 (2.50–4.22)
I am not at risk of getting an antibiotic-resistant infection, as long as I take my antibiotics correctly	405 (36.1)	**220** (**48.2)**	1.65 (1.32–2.05)

The table highlights associations that are statistically significant (*P* < 0.05) in relation to gender. A gender with a higher percentage of incorrect responses is indicated in bold.

**Table 3.** Incorrect KAPs related to AMR among different groups: age

KAPs	Age		
	18–34 *n* (%)	≥35 *n* (%)	OR (95 CI)
Cooking habits			
I wash my hands before cooking (No)	**62 (10.7)**	102 (8.4)	1.49 (1.06–2.07)
After handling raw food (meat and vegetables), I wash my hands with soap (No)	**118 (22.8)**	138 (11.5)	2.31 (1.76–3.03)
I use the same kitchen utensils for handling raw and ready-to-eat foods (Yes)	**205 (40.9)**	244 (20.2)	2.71 (2.16–3.4)
I wash kitchen utensils that have been used for raw food before using them to prepare other foods (No)	**123 (24.7)**	167 (15.8)	2.02 (1.55–2.62)
I wash fruits and vegetables before eating them (No)	**139 (27.5)**	222 (18.5)	1.68 (1.32–2.15)
I wash my hands before eating (No)	**127 (25.3)**	246 (20.8)	1.29 (1.01–1.65)
Antimicrobial use			
When you get a prescription for antibiotics, do you follow the recommended length of treatment and daily dosage? (No or sometimes)	**134 (26.7)**	110 (9.2)	3.6 (2.74–4.78)
Do you stop using the medication once symptoms start to disappear? (Sometimes or always)	**260 (53.7)**	225 (18.9)	4.97 (3.96–6.28)
Have you ever taken an oral antibiotic treatment (by mouth) without a medical prescription? (Yes)	**91 (20.6)**	124 (10.9)	2.12 (1.58–2.86)
Contact with animals			
I can exchange resistant bacteria with my pet (False)	**143 (30.6)**	265 (26.6)	1.22 (0.96–1.56)
The use of antibiotics in livestock and crops can increase the presence of resistant bacteria in the environment (False)	**110 (24.1)**	138 (12.4)	2.15 (1.63–2.84)
The use of antibiotics in livestock and crops can affect me directly (I can get resistant bacteria in my body) (False)			
Resistant bacteria are only found in hospitals (True)	**98 (20.4)**	91 (8.6)	2.98 (2.19–4.07)
General knowledge about AMR			
Antibiotic resistance occurs when your body becomes resistant to antibiotics and they no longer work as well (True)	387 (27.0)	**1045 (72.97)**	2.41 (1.79–3.23)
Many infections are becoming increasingly resistant to antibiotic treatment (False)	**99 (20.3)**	111 (9.4)	2.41 (1.79–3.24)
If bacteria are resistant to antibiotics, it can be very difficult or impossible to treat the infections they cause (False)	**123 (24.6)**	164 (15.2)	2.00 (1.54–2.60)
Antibiotic resistance is an issue that could affect me or my family (False)	**95 (22.0)**	107 (10.4)	2.42 (1.79–3.26)
Antibiotic resistance is an issue in other countries but not here (True)	**76 (15.6)**	93 (8.1)	2.17 (1.57–3.00)
Antibiotic resistance is only a problem for people who take antibiotics regularly (True)	**123 (25.3)**	183 (15.9)	1.85 (1.42–2.39)
Bacteria that are resistant to antibiotics can be spread from person to person (False)	**134 (30.6)**	212 (27.9)	1.65 (1.28–2.12)
Antibiotic-resistant infections could make medical procedures like surgery, organ transplants and cancer treatment much more dangerous (False)	**91 (24.8)**	112 (12.0)	2.25 (1.66–3.05)
Levels of awareness and understanding of how to combat AMR (strongly disagree or neither agree nor disagree)			
Farmers should give fewer antibiotics to food-producing animals	**215 (45.5)**	335 (29.2)	2.02 (1.62–2.53)
People should not keep antibiotics and use them later for other illnesses	**239 (48.2)**	242 (20.3)	3.65 (2.92–4.57)
Parents should make sure all of their children’s vaccinations are up-to-date	**216 (44.3)**	278 (23.8)	2.55 (2.04–3.18)
People should wash their hands regularly	**192 (39.0)**	214 (17.8)	2.95 (2.34–3.73)
Doctors should only prescribe antibiotics when they are needed	**180 (36.3)**	148 (12.3)	4.05 (3.15–5.21)
People should only use antibiotics when prescribed by a doctor or nurse	**185 (37.2)**	191 (15.7)	3.13 (2.47–3.98)
Everyone needs to use antibiotics responsibly	**160 (32.1)**	149 (12.4)	3.34 (2.59–4.31)
I am not at risk of getting an antibiotic-resistant infection, as long as I take my antibiotics correctly	236 (36.8)	**405 (63.2)**	1.67 (1.34–2.07)

The table highlights associations that are statistically significant (*P* < 0.05) in relation to age. An age group with a higher percentage of incorrect responses is indicated in bold.

**Table 4.** Incorrect KAPs related to AMR among different groups: education

KAPs	Education		
	Higher education *n* (%)	Others *n* (%)	OR (95 CI)
Cooking habits			
I wash my hands before cooking (No)	71 (44.1)	**90 (55.9)**	1.20 (0.86–1.66)
After handling raw food (meat and vegetables), I wash my hands with soap (No)	74 (29.2)	**180 (70.8)**	2.58 (1.94–3.46)
I use the same kitchen utensils for handling raw and ready-to-eat foods (Yes)	173 (38.7)	**274 (61.3)**	1.71 (1.37–2.13)
I wash kitchen utensils that have been used for raw food before using them to prepare other foods (No)	100 (36.4)	**187 (63.6)**	1.96 (1.51–2.56)
I wash fruits and vegetables before eating them (No)	131 (36.5)	**228 (63.5)**	1.86 (1.47–2.37)
I wash my hands before eating (No)	156 (41.9)	**216 (58.1)**	1.42 (1.12–1.79)
Antimicrobial use			
When you get a prescription for antibiotics, do you follow the recommended length of treatment and daily dosage? (No or sometimes)	45 (5.5)	**198 (23.0)**	**5.10 (3.66–7.25)**
Do you stop using the medication once symptoms start to disappear? (Sometimes or always)	153 (31.6)	**330 (68.3)**	**2.75 (2.20–3.44)**
Have you ever taken an oral antibiotic treatment (by mouth) without a medical prescription? (Yes)	100 (12.3)	**115 (15.0)**	1.25 (0.93–1.67)
Contact with animals			
I can exchange resistant bacteria with my pet (False)	155 (14.7)	**246 (63.9)**	1.72 (1.37–2.18)
The use of antibiotics in livestock and crops can increase the presence of resistant bacteria in the environment (False)	38 (13.1)	**207 (79.1)**	7.20 (5.07–10.51)
The use of antibiotics in livestock and crops can affect me directly (I can get resistant bacteria in my body) (False)	186 (34.1)	**267 (72.6)**	1.65 (1.32–2.07)
Resistant bacteria are only found in hospitals (True)	50 (9.8)	**137 (17.0)**	3.11 (2.22–4.40)
General knowledge about AMR			
Antibiotic resistance occurs when your body becomes resistant to antibiotics and they no longer work as well (True)	455 (58.3)	**554 (66.5)**	1.50 (1.23–1.84)
Many infections are becoming increasingly resistant to antibiotic treatment (False)	39 (4.9)	**173 (20.9)**	5.05 (3.55–7.36)
If bacteria are resistant to antibiotics, it can be very difficult or impossible to treat the infections they cause (False)	69 (8.7)	**217 (26.4)**	3.78 (2.84–5.09)
Antibiotic resistance is an issue that could affect me or my family (False)	27 (3.4)	**173 (23.4)**	7.69 (5.14–11.94)
Antibiotic resistance is an issue in other countries but not here (True)	39 (4.9)	**126 (15.2)**	3.48 (2.42–5.13)
Antibiotic resistance is only a problem for people who take antibiotics regularly (True)	99 (12.5)	**200 (24.2)**	2.32 (1.78–3.03)
Bacteria that are resistant to antibiotics can be spread from person to person (False)	84 (10.4)	**259 (34.5)**	4.05 (3.10–5.32)
Antibiotic-resistant infections could make medical procedures like surgery, organ transplants and cancer treatment much more dangerous (False)	36 (4.1)	**165 (20.8)**	5.32 (3.70–7.87)
Levels of awareness and understanding of how to combat AMR (strongly disagree or neither agree nor disagree)			
Farmers should give fewer antibiotics to food-producing animals	183 (23.88)	**358 (42.28)**	**2.45 (1.98–3.05)**
People should not keep antibiotics and use them later for other illnesses	163 (17.65)	**313 (30.34)**	**2.25 (1.81–2.82)**
Parents should make sure all of their children’s vaccinations are up-to-date	173 (16.24)	**314 (28.26)**	**2.16 (1.74–2.69)**
People should wash their hands regularly	129 (12.37)	**272 (19.81)**	**2.49 (1.97–3.16)**
Doctors should only prescribe antibiotics when they are needed	66 (5.87)	**260 (20.63)**	**4.97 (3.74–6.69)**
People should only use antibiotics when prescribed by a doctor or nurse	87 (7.64)	**284 (23.38)**	**4.08 (3.15–5.34)**
Everyone needs to use antibiotics responsibly	**65 (18.99)**	243 (13.16)	**4.67 (3.50–6.30)**
I am not at risk of getting an antibiotic-resistant infection, as long as I take my antibiotics correctly	211 (26.28)	**422 (51.53)**	**2.86 (2.32–3.52)**

The table highlights associations that are statistically significant (*P* < 0.05) in relation to education level. A group with a higher percentage of incorrect responses is indicated in bold.

**Table 5.** Incorrect KAPs related to AMR among different groups: field of work

KAPs	Field of work/study		
	Health and science *n* (%)	Other fields *n* (%)	OR (95 CI)
Cooking habits			
I wash my hands before cooking (No)	42 (25.1)	**125 (74.9)**	1.52 (1.06–2.21)
After handling raw food (meat and vegetables), I wash my hands with soap (No)	62 (23.1)	**205 (76.9)**	1.76 (1.30–2.40)
I use the same kitchen utensils for handling raw and ready-to-eat foods (Yes)	109 (23.6)	**349 (76.4)**	1.83 (1.44–2.34)
I wash kitchen utensils that have been used for raw food before using them to prepare other foods (No)	78 (29.7)	**220 (70.3)**	1.46 (1.11–1.94)
I wash my hands before eating (No)	139 (36.6)	**243 (63.4)**	1.19 (0.94–1.51)
Antimicrobial use			
When you get a prescription for antibiotics, do you follow the recommended length of treatment and daily dosage? (No or sometimes)	52 (8.54)	**193 (18.45)**	2.00 (1.46–2.79)
Do you stop using the medication once symptoms start to disappear? (Sometimes or always)	86 (17.20)	**405 (49.20)**	3.04 (2.36–3.97)
Contact with animals			
I can exchange resistant bacteria with my pet (False)	91 (12.41)	**320 (43.65)**	2.22 (1.71–2.90)
The use of antibiotics in livestock and crops can increase the presence of resistant bacteria in the environment (False)	40 (5.18)	**220 (28.33)**	3.59 (2.54–5.19)
The use of antibiotics in livestock and crops can affect me directly (I can get resistant bacteria in my body) (False)	136 (13.61)	**330 (86.39)**	1.46 (1.15–1.85)
Resistant bacteria are only found in hospitals (True)	28 (4.99)	**164 (95.01)**	3.33 (2.24–4.68)
General knowledge about AMR			
Antibiotic resistance occurs when your body becomes resistant to antibiotics and they no longer work as well (True)	290 (43.8)	**744 (67.5)**	1.98 (1.61–2.42)
Many infections are becoming increasingly resistant to antibiotic treatment (False)	31 (5.5)	**191 (17.4)**	3.58 (2.45–5.40)
If bacteria are resistant to antibiotics, it can be very difficult or impossible to treat the infections they cause (False)	49 (8.7)	**248 (18.1)**	3.10 (2.26–4.34)
Antibiotic resistance is an issue that could affect me or my family (False)	35 (6.3)	**174 (16.1)**	2.85 (1.98–4.23)
Antibiotic resistance is an issue in other countries but not here (True)	20 (3.5)	**155 (14.1)**	4.48 (2.84–7.48)
Antibiotic resistance is only a problem for people who take antibiotics regularly (True)	68 (11.6)	**243 (23.1)**	2.16 (1.62–2.99)
Bacteria that are resistant to antibiotics can be spread from person to person (False)	55 (10.5)	**303 (30.7)**	3.76 (2.78–5.18)
Antibiotic-resistant infections could make medical procedures like surgery, organ transplants and cancer treatment much more dangerous (False)	37 (6.6)	**176 (16.8)**	2.84 (1.98–4.18)
Levels of awareness and understanding of how to combat AMR (strongly disagree or neither agree nor disagree)			
Farmers should give fewer antibiotics to food-producing animals	151 (27.7)	**404 (37.1)**	1.52 (1.22, 1.91)
People should not keep antibiotics and use them later for other illnesses	91 (16.1)	**404 (25.7)**	2.87 (2.23, 3.72)
Parents should make sure all of their children’s vaccinations are up-to-date	119 (21.3)	**386 (34.6)**	1.96 (1.55–2.49)
People should wash their hands regularly	99 (19.71)	**316 (20.12)**	1.83 (1.43–2.37)
Doctors should only prescribe antibiotics when they are needed	52 (10.14)	**286 (10.17)**	0.30 (0.22–0.54)
People should only use antibiotics when prescribed by a doctor or nurse	72 (12.69)	**315 (25.13)**	2.61 (1.98–3.47)
Everyone needs to use antibiotics responsibly	53 (9.23)	**268 (23.37)**	3.0 (2.21- 4.15)
I am not at risk of getting an antibiotic-resistant infection, as long as I take my antibiotics correctly	164 (12.26)	**489 (23.99)**	2.0(1.61- 2.50)

The table highlights associations that are statistically significant (*P* < 0.05) in relation to field of work/study. A group with a higher percentage of incorrect responses is indicated in bold.

Compared with women, men were twice as likely not to wash their hands with soap after handling raw food [odds ratio (OR) 1.8, confidence interval (CI) 1.4–2.5] and nearly three times more likely to use the same kitchen utensils for raw and ready-to-eat foods (OR 2.7, CI 2.1–3.3). Men also had higher odds of stopping medication once symptoms disappeared (OR 4.31, CI 3.4–5.4) and of taking antibiotics without a prescription (OR 2.1, CI 1.6–2.9). Men were also more than three times more likely to believe that resistant bacteria were found only in hospitals (OR 3.4, CI 2.5–4.8). However, the misconception that antibiotic resistance occurs when one's body becomes resistant to drugs was more common among women (OR 1.3, CI 1.1–1.6). Comparisons across different age groups revealed that younger participants (18–34 years) were more likely to hold misconceptions and engage in risky behaviours related to AMR. Specifically, 40.8% of them used the same kitchen utensils for handling raw and ready-to-eat foods, and 53.7% stopped using medication once symptoms disappeared. On the other hand, participants older than 55 years demonstrated better practices and behaviours overall, with lower misconception rates in most categories. Yet, a notable proportion (46.4%) mistakenly believed that resistance occurs when the body becomes resistant to antibiotics.

Individuals who had completed undergraduate or graduate studies were less likely to hold erroneous beliefs, demonstrating greater adherence to recommended antimicrobial treatment courses and knowledge that bacteria resistant to antibiotics could spread from person to person and make medical procedures more dangerous. Respondents in health and science fields demonstrated better hygiene practices and adherence to antibiotic courses than did those in non-health fields.

## Discussion

Our study results showed that, in a diverse cohort from urban areas in the province of Quebec, AMR was a concept that was known to most participants, but where education initiatives still have a role. Our cohort provided a fair representation of the Quebec's general population. According to the 2021 Canadian National Census, the 25–34- and 35–44-year age groups collectively constituted 28.1% of Quebec's population, like our cohort. Nonetheless, women were overrepresented in our study (69.2%) when compared to Quebec's general population (50.4%). The high educational attainment among our participants represents Quebec's broader educational landscape, where 60.4% of the population 15 years and older held a postsecondary certificate, diploma, or degree, and 32.8% had a bachelor's degree or higher. Our sample's household composition also mirrors the characteristics prevalent in Quebec.

### Antimicrobials are still used without prescription, for suboptimal duration

Our results are similar to the WHO's Multi-country Awareness Report,^16^ where 19% of respondents used antibiotics without a prescription. This pattern varied across countries, with lower rates in South Africa (7%), Mexico (8%), Barbados (9%), Sudan (9%) and India (10%). Notably, the Russian Federation presented a significantly higher rate, with 44% of participants acquiring antibiotics without a prescription.^16^

Countries surveyed by the WHO included those where over-the-counter antibiotic sales are more prevalent, making higher rates of non-prescribed usage somewhat expected. However, our survey showed that this practice occurred in Canada, which is intriguing, given the country’s stringent regulations regarding antibiotic sales.^23^ This implies there might be overlooked or unregulated channels through which antibiotics can be obtained without proper medical oversight. Additionally, difficulties in accessing healthcare services might have led individuals to keep antibiotics for future use, particularly in cases of recurrent infections. In Canada, limited access to healthcare services, especially in remote or underserved communities, has been associated with the tendency to store antibiotics for future use or to share prescriptions.^24^ Another possible explanation for this finding is a misinterpretation of our survey question, with some respondents alluding to the original place of procurement rather than the actual source for their non-prescribed treatment. We hypothesize that leftover antibiotics from prior prescriptions are being repurposed for later self-medication. This theory gained credence from the significant proportion of respondents who also cited their home medicine cabinets as a source for non-prescribed antimicrobials (28.5%, *n* = 63).

Furthermore, it is worthwhile to explore the role of pharmacists, who are now authorized to prescribe antibiotics under specific circumstances. This factor could partially explain why some respondents identified pharmacies as their primary sources of non-prescribed antibiotics, perhaps not being aware of pharmacists’ prescription authority in these cases.

Thoroughly examining unsanctioned access to antimicrobials in Canada is imperative.^25^ Future studies could benefit from formulating questions specifically regarding the procurement of antimicrobials, particularly in the context of self-medication, to achieve a deeper understanding and more accurate interpretation of the data.

### Levels of awareness

Compared to prior studies, our findings indicated a higher level of awareness in our cohort about general knowledge of AMR and its impact.^16,21,26^ While some of these investigations were conducted in countries with healthcare systems that differ substantially from Canada's, they used questionnaires derived from the WHO survey, allowing meaningful comparisons with our findings.

Although most participants incorrectly attributed resistance to human immunity, this has also been reported in Nigeria (75.1%),^26^ and in the WHO survey (76%). This misconception is particularly concerning because individuals may perceive AMR as a non-transmissible condition and consequently neglect critical hygiene and preventive measures.^21^ In our study, we also examined varying levels of understanding regarding key terms related to AMR. Although the term ‘antimicrobial resistance’ is commonly used in academic circles,^27^ ‘antibiotic resistance’ and ‘antibiotic-resistant bacteria’ appeared to resonate more with the public and may be more effective in awareness campaigns to enhance engagement and understanding.

In our study, most participants did not perceive AMR as one of the greatest problems the world faces or express concern about its impact on themselves and their families. These results contrasted with those reported by Tangcharoensathien *et al*.,^28^ who reported substantially greater concern about AMR among participants. This difference may be explained by the fact that mortality associated with infections caused by AMR is not as prevalent in Canada.^29,30^

### Habits and perceptions

In the context of hygiene practices related to food preparation and consumption, our study uncovered some concerning trends, especially regarding the handling of raw foods. While home cooking can enhance diet quality, it can also serve as a potential source of foodborne illnesses due to improper hygiene practices, contributing to the spread of antimicrobial-resistant bacteria.^31^ In Canada, the spread of AMR in the food chain is a significant public health concern, with studies highlighting the presence of resistant bacteria in various food products and their potential to cause difficult-to-treat infections.^32,33^

In addition, some respondents did not recognize the risk of transmission of resistant bacteria between animals and humans. This finding aligns with previous studies from the USA where between 24.2% and 57% of participants were unaware of the risk of transmitting resistant bacteria between animals and humans.

### Ambivalence towards AMR mitigation strategies

Here, individuals were surveyed on their perspectives regarding various strategies and policies to mitigate AMR. Respondents who selected ‘neither agreed nor disagreed’ represented individuals with uncertain or ambivalent views, highlighting opportunities for targeted educational interventions.^34,35^ In our survey, uncertainty was particularly evident regarding vaccination and the use of antimicrobials in crop cultivation and animal production. These findings contrasted with those reported by Muflih *et al*.,^36^ which indicated elevated awareness among participants, who reported higher awareness regarding the importance of childhood vaccination (82.9%) and the appropriate prescription of antibiotics by healthcare professionals (82%).

### Targeted education might improve AMR-related behaviours

According to our univariate and multivariate analysis (Tables 1–6), men and younger individuals are more likely to engage in risky AMR-related behaviours, while higher education and health-related professions are correlated with better AMR knowledge and practices, a tendency verified in other KAP AMR-related studies,^28,36–39^ which can be explained by multiple factors, including health literacy, educational background and prior exposure to healthcare settings.

**Table 6.** Multivariate analyses for selected KAPs and variables (gender, age, education and field of work)

KAPs	Predictor	Coefficient	OR	95% CI	*P* value
I wash my hands before cooking (No)	Intercept	−2.29	0.1	(0.07–0.14)	<0.05
	Age	−0.34	0.72	(0.51–1.00)	<0.05
	Field of work	0.43	1.53	(1.08–2.22)	<0.05
After handling raw food (meat and vegetables), I wash my hands with soap (No).	Intercept	−2.13	0.12	(0.08–0.17)	<0.05
	Gender	−0.27	0.76	(0.56–1.04)	0.08
	Age	0.89	2.44	(1.82–3.23)	<0.05
	Education	−0.8	0.45	(0.33, 0.60)	<0.05
	Field of work	−0.49	0.61	(0.44–0.85)	<0.05
I use the same kitchen utensils for handling raw and ready-to-eat foods (Yes).	Intercept	1.17	3.22	(2.44–4.28)	<0.05
	Gender	−0.68	0.51	(0.40–0.65)	<0.05
	Age	0.99	2.7	(2.12–3.44)	<0.05
	Education	−0.39	0.68	(0.53–0.85)	<0.05
	Field of work	−0.5	0.6	(0.46–0.78)	<0.05
I wash kitchen utensils that have been used for raw food before using them to prepare other foods (No).	Intercept	−1.7	0.18	(0.13–0.25)	<0.05
	Gender	0.12	1.13	(0.84–1.51)	0.4112
	Age	−0.7	0.5	(0.38–0.66)	<0.05
	Education	0.58	1.79	(1.36–2.35)	<0.05
	Field of work	0.28	1.32	(0.98–1.79)	0.07
I wash fruits and vegetables before eating them (No)	Intercept	−1.46	0.23	(0.18–0.30)	<0.05
	Gender	0.19	1.2	(0.92–1.56)	0.1661
	Age	−0.45	0.64	(0.50–0.83)	<0.05
	Education	0.63	1.88	(1.47–2.42)	<0.05
I wash my hands before eating (No)	Intercept	−1.13	0.32	(0.24–0.42)	<0.05
	Age	−0.23	0.79	(0.62–1.02)	0.06
	Education	0.36	1.43	(1.13–1.81)	<0.05
	Field of work	−0.24	0.78	(0.62–1.00)	<0.05
When you get a prescription for antibiotics, do you follow the recommended length of treatment and daily dosage? (No and sometimes)	Intercept	−2.55	0.08	(0.05–0.12)	<0.05
	Gender	0.41	1.5	(1.09–2.07)	<0.05
	Age	−1.31	0.27	(0.20–0.36)	<0.05
	Education	1.62	5.03	(3.54–7.30)	<0.05
Have you ever taken an oral antibiotic treatment (by mouth) without a medical prescription? (Yes)	Intercept	1.74	5.67	(4.11–7.96)	<0.05
	Gender	−0.55	0.58	(0.42–0.80)	<0.05
	Age	0.71	2.03	(1.48–2.77)	<0.05
	Field of work	−0.26	0.77	(0.55–1.07)	0.12
I can exchange resistant bacteria with my pet (False)	Intercept	−1.72	0.18	(0.13–0.24)	<0.05
	Gender	0.41	1.51	(1.17–1.96)	<0.05
	Age	−0.17	0.84	(0.65–1.09)	<0.05
	Education	0.47	1.61	(1.26–2.05)	<0.05
	Field of work	0.7	2.02	(1.54–2.68)	<0.05
The use of antibiotics in livestock and crops can increase the presence of resistant bacteria in the environment (False)	Intercept	−3.39	0.03	(0.02–0.05)	<0.05
	Gender	0.6	1.83	(1.33–2.52)	<0.05
	Age	−0.83	0.44	(0.32–0.60)	<0.05
	Education	1.88	6.56	(4.55–9.68)	<0.05
	Field of work	1.12	3.07	(2.09–4.61)	<0.05
The use of antibiotics in livestock and crops can affect me directly (I can get resistant bacteria in my body) (False)	Intercept	−1.2	0.3	(0.23–0.40)	<0.05
	Age	−0.06	0.94	(0.74–1.21)	0.64
	Education	0.48	1.61	(1.28–2.02)	<0.05
	Field of work	0.33	1.39	(1.09–1.77)	<0.05
Resistant bacteria are only found in hospitals (True)	Intercept	3.1	22.18	(13.84–37.11)	<0.05
	Gender	−0.77	0.46	(0.33–0.65)	<0.05
	Age	1.13	3.1	(2.21–4.35)	<0.05
	Education	−0.97	0.38	(0.26–0.54)	<0.05
	Field of work	−1.15	0.32	(0.20–0.49)	<0.05
Antibiotic resistance occurs when your body becomes resistant to antibiotics and they no longer work as well (True)	Intercept	0.42	1.51	(1.18–1.95)	<0.05
	Gender	0.37	1.45	(1.14–1.85)	<0.05
	Age	−0.55	0.58	(0.46–0.72)	<0.05
	Education	−0.41	0.67	(0.54–0.82)	<0.05
	Field of work	−0.67	0.51	(0.41–0.64)	<0.05
Many infections are becoming increasingly resistant to antibiotic treatment (False)	Intercept	−3.33	0.04	(0.02–0.06)	<0.05
	Gender	0.53	1.71	(1.22–2.38)	<0.05
	Age	−0.88	0.41	(0.30–0.57)	<0.05
	Education	1.42	4.12	(2.84–6.11)	<0.05
	Field of work	1.08	2.95	(1.94–4.64)	<0.05
If bacteria are resistant to antibiotics, it can be very difficult or impossible to treat the infections they cause (False)	Intercept	−2.88	0.06	(0.04–0.08)	<0.05
	Gender	0.7	2.02	(1.50–2.70)	<0.05
	Age	−0.63	0.53	(0.40–0.72)	<0.05
	Education	1.23	3.42	(2.52–4.70)	<0.05
	Field of work	0.94	2.57	(1.81–3.72)	<0.05
Antibiotic resistance is an issue that could affect me or my family (False)	Intercept	−3.7	0.02	(0.01–0.04)	<0.05
	Gender	0.85	2.34	(1.66–3.30)	<0.05
	Age	−0.84	0.43	(0.31–0.61)	<0.05
	Education	1.91	6.77	(4.42–10.77)	<0.05
	Field of work	0.81	2.26	(1.48–3.52)	<0.05
Antibiotic resistance is an issue in other countries but not here (True)	Intercept	3.84	46.45	(26.28–87.82)	<0.05
	Gender	−0.86	0.42	(0.30–0.60)	<0.05
	Age	0.8	2.22	(1.55–3.17)	<0.05
	Education	−1.04	0.35	(0.23–0.52)	<0.05
	Field of work	−1.48	0.23	(0.13–0.38)	<0.05
Antibiotic resistance is only a problem for people who take antibiotics regularly (True)	Intercept	2.14	8.52	(6.06–12.20)	<0.05
	Gender	−0.67	0.51	(0.39–0.68)	<0.05
	Age	0.64	1.9	(1.44–2.52)	<0.05
	Education	−0.77	0.46	(0.35–0.61)	<0.05
	Field of work	−0.67	0.51	(0.37–0.70)	<0.05
Bacteria that are resistant to antibiotics can be spread from person to person (False)	Intercept	−2.6	0.07	(0.05–0.11)	<0.05
	Gender	0.41	1.5	(1.13–1.99)	<0.05
	Age	−0.53	0.59	(0.44–0.79)	<0.05
	Education	1.22	3.39	(2.56–4.52)	<0.05
	Field of work	1.17	3.23	(2.32–4.56)	<0.05
Antibiotic-resistant infections could make medical procedures like surgery, organ transplants and cancer treatment much more dangerous (False)	Intercept	−3.29	0.04	(0.02–0.06)	<0.05
	Gender	0.71	2.03	(1.44–2.85)	<0.05
	Age	−0.74	0.47	(0.34–0.67)	<0.05
	Education	1.52	4.59	(3.11–6.94)	<0.05
	Field of work	0.73	2.07	(1.39–3.16)	<0.05
Farmers should give fewer antibiotics to food-producing animals (Strongly disagree and neither agree nor disagree)	Intercept	1.68	5.35	(3.13–9.48)	<0.05
	Gender	−0.66	0.52	(0.34–0.78)	<0.05
	Age	0.37	1.44	(0.96–2.16)	0.08
	Education	−0.58	0.56	(0.35–0.88)	<0.05
	Field of work	−0.31	0.74	(0.45–1.18)	0.21
People should not keep antibiotics and use them later for other illnesses (Strongly disagree and neither agree nor disagree)	Intercept	0.88	2.41	(1.41–4.24)	<0.05
	Gender	0.21	1.23	(0.83–1.84)	0.3
	Age	−0.28	0.76	(0.51–1.12)	0.16
	Education	−0.12	0.89	(0.58–1.34)	0.57
	Field of work	−0.12	0.89	(0.52–1.47)	0.64
Parents should make sure all of their children’s vaccinations are up-to-date (Strongly disagree and neither agree nor disagree)	Intercept	0.89	2.44	(1.46–4.15)	<0.05
	Gender	−0.37	0.69	(0.46–1.03)	0.07
	Age	0	1	(0.68–1.47)	0.999
	Education	−0.25	0.78	(0.52–1.17)	0.23
	Field of work	−0.17	0.85	(0.53–1.33)	0.47
People should wash their hands regularly (Strongly disagree and neither agree nor disagree)	Intercept	0.99	2.7	(1.53–4.90)	<0.05
	Gender	−0.11	0.9	(0.57–1.42)	0.64
	Age	0.1	1.1	(0.72–1.70)	0.66
	Education	−0.45	0.64	(0.39–1.02)	0.07
	Field of work	−0.08	0.93	(0.55–1.54)	0.77
Doctors should only prescribe antibiotics when they are needed (Strongly disagree and neither agree nor disagree)	Intercept	0.99	2.68	(1.22–6.14)	<0.05
	Gender	−0.4	0.67	(0.42–1.07)	0.1
	Age	−0.11	0.9	(0.56–1.44)	0.65
	Education	−0.43	0.65	(0.35–1.17)	0.16
	Field of work	0.09	1.09	(0.57–2.06)	0.79
People should only use antibiotics when prescribed by a doctor or nurse (Strongly disagree)	Intercept	1.13	3.1	(1.52–6.59)	<0.05
	Gender	0.08	1.08	(0.69–1.70)	0.72
	Age	0.01	1.01	(0.65–1.56)	0.98
	Education	−0.39	0.68	(0.39–1.14)	0.15
	Field of work	−0.32	0.72	(0.39–1.29)	0.28
Antibiotic resistance is one of the biggest problems the world faces (Strongly disagree)	Intercept	1.53	4.6	(2.94–7.35)	<0.05
	Gender	−0.49	0.61	(0.43–0.88)	<0.05
	Age	−0.06	0.94	(0.66–1.33)	0.72
	Education	−0.62	0.54	(0.38–0.76)	<0.05
	Field of work	−0.19	0.82	(0.57–1.19)	0.31
Medical experts will solve the problem of antibiotic resistance before it becomes too serious (Strongly agree)	Intercept	1.54	4.65	(2.96–7.49)	<0.05
	Gender	−0.38	0.69	(0.47–0.99)	<0.05
	Age	0.07	1.08	(0.75–1.53)	0.68
	Education	−0.29	0.75	(0.52–1.07)	0.11
	Field of work	−0.42	0.65	(0.44–0.97)	<0.05
Everyone needs to use antibiotics responsibly (Strongly disagree)	Intercept	−1.6	0.2	(0.14–0.29)	<0.05
	Gender	0.77	2.15	(1.61–2.88)	<0.05
	Age	0	1	(0.75–1.35)	0.99
	Education	0.12	1.12	(0.86–1.48)	0.4
	Field of work	0.57	1.76	(1.29–2.43)	<0.05
People like me can't do much to stop antibiotic resistance (Strongly agree)	Intercept	−1.41	0.24	(−1.88 to −0.94)	<0.05
	Gender	0.18	1.19	(0.86–1.65)	0.28
	Age	0.19	1.21	(0.87–1.68)	0.26
	Education	0.24	1.28	(0.95–1.73)	0.11
	Field of work	0.39	1.48	(1.04–2.12)	<0.05
I am worried about the impact that antibiotic resistance will have on my health and that of my family (Strongly disagree)	Intercept	−1.41	0.24	(0.16–0.37)	<0.05
	Gender	0.18	1.19	(0.86–1.65)	0.28
	Age	0.19	1.21	(0.87–1.68)	0.26
	Education	0.24	1.28	(0.95–1.73)	0.11
	Field of work	0.39	1.48	(1.04–2.12)	<0.05
I am not at risk of getting an antibiotic-resistant infection, as long as I take my antibiotics correctly (Strongly agree)	Intercept	1.48	4.39	(2.92–6.72)	<0.05
	Gender	0.31	0.74	0.53–1.03)	0.073
	Age	0.09	1.1	(0.79–1.52)	0.58
	Education	−0.69	0.5	(0.36–0.69)	0.05
	Field of work	−0.39	0.68	(0.47–0.96)	0.032

This table presents the results of logistic regression analyses examining various hygiene and health-related practices (KAPs) among different demographic groups. The predictors include gender (men versus women), age (18–34 versus 35+ years), education (higher education—bachelor’s degree or above—versus other) and field of work (health and science versus other fields). The table displays the intercept, coefficient, odds ratio (OR), 95% CI and *P* value for each predictor. The model selection was based on the Akaike Information Criterion to ensure the best fit.

## Strengths and limitations

A major strength of this study is the use of a comprehensive WHO-standardized questionnaire addressing key AMR-related KAPs. Additionally, the survey’s anonymous and voluntary design is likely to encourage participants to provide genuine responses, thereby minimizing the likelihood of receiving socially desirable answers.

This study had several limitations that must be acknowledged. A primary constraint is the reliance on self-reported data, inherently subject to information and recall biases, which could impact the accuracy of the collected data. Another limitation is the cross-sectional design of the study.

Furthermore, the representativity of the sample, due to the adoption of a convenient sampling strategy, constitutes a significant limitation of this study. In our sample, most participants were women, and many were from the health and sciences sectors, likely reflecting the survey's distribution methods. Primarily circulated on social media and through electronic mailing lists of student associations, university departments, and research centres, the survey might have predominantly reached these specific demographics.

Despite these limitations, the study's engagement with a large cohort of 1774 individuals from the general population of Quebec is a notable strength. While this group may not completely represent the entire demographic spectrum of Quebec's population, its size and diversity allowed us to capture a broad range of perspectives and identify important knowledge and practice gaps related to AMR.

## Key findings and recommendations

In this investigation, we aimed to explore the KAPs about AMR in a diverse and representative cohort in Quebec, Canada. Our results revealed that although participants displayed a fair knowledge of AMR, there were several points that urge improvement.

Given the study’s insights, the following could improve awareness: (i) enhanced public education, using targeted campaign based on knowledge gaps—starting with men, younger individuals and those with lower education levels; () investigate and address loopholes in the prescription and sale of antimicrobials, which requires comprehensive surveillance data on antimicrobial purchase and usage; (iii) emphasize the dangers of non-prescribed antibiotic use, including the repurposing of previously prescribed antibiotics without medical guidance; (iv) use terms such as ‘antibiotic resistance’ in public campaigns for better resonance with the general population; (v) promote AMR education from an early age by integrating it into secondary school curricula and (vi) encourage public participation in AMR mitigation strategies, emphasizing community roles in health policy development.

Future research should explore the effectiveness of interventions and investigate new strategies to further enhance public understanding of and response to AMR.

## Supplementary Material

dlag162_Supplementary_Data

## Data Availability

The datasets and materials supporting the conclusions of this study are available as Supplementary Files accompanying this manuscript. These include the Data Dictionary (an Excel workbook that enumerates the variables with their definitions, formats, and explanations), the Complete Database (a comprehensive Excel workbook containing all collected data, including text-based variables), and the Simplified Database (a streamlined version of the dataset excluding text-based variables). All datasets have been anonymized to ensure participant confidentiality. Additionally, the research questionnaires used in the study are provided in both English and French, detailing the survey layout and questions. The R script (Script_Article1.R) used for data processing, analysis, and visualization is also included to facilitate transparency and reproducibility. Further inquiries regarding the data or materials can be directed to the corresponding author.

## Supplementary data

Figures S1–S7 and Table S1 are available as Supplementary data at *JAC-AMR* Online.
