# Supplementary material for: Mapping antimicrobial resistance awareness in Canada: what are our knowledge gaps?
Source: JAC Antimicrob Resist. 2026 Aug 26;8(4):dlag162. doi: 10.1093/jacamr/dlag162 (PMC13511133; doi:10.1093/jacamr/dlag162)
Supplement: Supplementary file 1 [file dlag162_supplementary_data.zip › Supplementary_Materials.docx]

**Supplementary Materials**

**Supplementary Text S1. Social Media Campaign Reach and Demographic Profile.** Social media platforms were instrumental in targeting a broad audience, with a campaign's reach most pronounced on Facebook, with 92,610 impressions, resulting in 3,304 link clicks. The Audience Network also played a significant role, exposing 19,150 individuals and accumulating 503 link clicks. Despite a lower reach of 3,330 impressions, Instagram generated 106 link clicks. Demographically, impressions of the survey were mostly seen in women (64.6%, *n*= 184,671) and in those 65 years and above (51.9%, *n*=148,508).

**Supplementary Text S2.** **Detailed statistical analysis.** To assess statistical significance in univariate analyses, we applied Yates' correction to chi-square tests when expected frequencies were close to, but not below, five, to reduce the risk of Type I errors. When expected frequencies were below five, we used Fisher's exact test. For multivariable analyses, we included propositions for which significant differences in misconception frequencies were observed between demographic groups (e.g., gender, educational level, and field of work), together with variables identified a priori as important confounders. We assessed model fit using the Akaike Information Criterion (AIC) and retained the most parsimonious model. The outcome of each logistic regression model was the likelihood of holding a misconception for a specific statement. To facilitate interpretation from the perspective of misconception likelihood, Odds Ratios (ORs) below 1 were inverted. Consequently, higher ORs consistently indicate a greater likelihood of misconception. Because misconception frequencies varied across questions, ORs should not be compared directly between different statements.

**Table S1. Geographic Distribution of Survey Participants in Québec (not including Montreal and Laval).** This table enumerates the participants from various cities across Québec. 'Other cities' encapsulate a significant portion of respondents, while individual cities such as Longueuil, Repentigny, and Saint Jerome also have notable representations. The table is organized by descending order of participant count and provides both the number of participants and their corresponding percentage of the total sample size (*n*=1774).

**Table S2. Univariate Analysis of Factors Associated with Misconceptions Regarding AMR.** This extensive table outlines the relationship between various factors and misconceptions about antimicrobial resistance (AMR), segmented by demographic categories including gender, nationality, age groups, education, and field of work or study. For each factor, odds ratios (OR) and 95% confidence intervals (CI) are provided to evaluate the strength of association. Data indicating statistically significant associations are color-highlighted for quick reference.

**Figure S1. Participant Selection Flowchart.** The flowchart delineates the participant screening and inclusion process. The initial cohort comprised 2081 respondents, for an overall survey completion rate of 87.5%. Of these, 1821 participants submitted complete responses. The exclusion criteria were applied at successive stages: residency outside Quebec (21 excluded), prior participation in the survey (5 excluded), and having another household member who had already completed the questionnaire (2 excluded). Additionally, 19 respondents were excluded because they selected "I prefer not to answer" for all items or because they provided responses that were inconsistent or logically incongruent. The final sample in the present study included 1774 participants. The flowchart uses standard visual indicators, and the final number included is highlighted in green.

**Figure S2.** **Awareness of AMR terms**. This bar chart presents survey data on participant awareness of various antibiotic resistance-related terms. The bars are color-coded to indicate responses: blue for 'Yes', indicating awareness; yellow for 'No', indicating a lack of awareness; and gray for 'I don't know/prefer not to answer'. The chart includes responses to terms such as 'Antibiotic Resistance', 'Superbugs', 'Antimicrobial Resistance', 'Drug resistance', and 'Antibiotic-resistant bacteria'. The horizontal bars are proportionally sized to represent the percentage of respondents aware of each term.

**Figure S3. Sources of Information on AMR**. This bar chart illustrates the sources from which participants have heard about the AMR terms detailed in Figure S2. The color-coding for the sources is as follows: 'Doctor or nurse' is represented in blue, 'Pharmacist' in orange, 'Family member or friend' in green, 'Internet (including social media)' in purple, 'Traditional media (newspaper, TV, radio)' in yellow, 'Specific campaign' in red, 'Other' in pink, and 'Can't remember' in gray.

**Figure S4. Public awareness of antibiotic resistance related to pets, livestock, and crops (*n*=1774).** The chart illustrates participants’ knowledge, attitudes, and practices (KAPs) related to antimicrobial resistance (AMR) across the One Health spectrum (animal and environmental domains). Beliefs are listed along the vertical axis, with corresponding response percentages along the horizontal axis. The blue bars indicate the proportion of participants acknowledging the statements as true. The yellow bars denote 'False' responses, while the gray bars represent individuals who were uncertain or opted not to respond. Red arrows underscore the percentage of incorrect beliefs.

**Figure S5.** **Survey Responses on Pet Ownership and Antibiotic Use.** This figure consists of two panels depicting the results of a survey about pet ownership and the administration of antibiotics. Panel A shows the responses to "Do you have animals?" with color coding: blue for 'Yes', yellow for 'No', and gray for 'I prefer not to answer'. Panel B presents the answers to "If so, do you give them antibiotics?" with the same respondents who answered 'Yes' in Panel A. The color code is as follows: yellow for 'No', blue for 'Yes, just to treat infections', red for 'Yes, frequently', and gray for 'I don’t know/I prefer not to answer'.

**Figure S6. Self-reported Cooking and Eating Hygiene Practices (*n*=1774).** The bar chart presents the percentage of participants who reportedly adhered or did not adhere to specific cooking and hygiene practices. The listed practices are shown on the vertical axis, with the corresponding percentages of responses displayed along the horizontal axis. The blue bars represent 'Yes' responses, indicating compliance with recommended practices, while the yellow bars signify 'No' responses, generally reflecting noncompliance — except for the third item, where 'No' indicates the correct behavior of not using the same utensils for raw and ready-to-eat foods. The gray bars denote respondents who were uncertain or chose not to answer. The red arrows emphasize the proportion of participants engaging in incorrect practices.

**Figure S7. Antimicrobial Resistance Magnitude and Personal Impact (*n*=1774).** The bar chart shows participants’ perceptions of the magnitude of AMR and its impact on them personally. Statements are listed along the vertical axis, with corresponding response percentages along the horizontal axis. Responses are segmented by the degree of agreement: 'Strongly agree' (blue); 'Neither agree nor disagree' (orange); 'Strongly disagree' (yellow); and 'I do not know/prefer not to answer' (gray). Arrows signify incorrect perceptions or neutrality.

**Data_Dictionary.xlsx:** This Excel workbook enumerates the variables with their respective definitions, formats, and detailed explanations, facilitating a comprehensive understanding of the data’s structure.

**Database_complete.xlsx:** This comprehensive Microsoft Excel workbook includes our full dataset, including text-based variables.

**Database_simplified.xlsx:** This Excel workbook is a streamlined version of the complete database, containing all variables except those in a text-based format.

**Research_Questionnaires.docx:** This Microsoft Word document contains the questionnaires used in this research, detailing the questions and the layout of the survey (English and French versions).

**Script_Article1.R:** The R programming language script file encompassing the code used for processing, analyzing, and visualizing the study’s data.
